# Supplementary material for: Development of New Restorer Lines Carrying Some Restoring Fertility Genes with Flowering, Yield and Grains Quality Characteristics in Rice (Oryza sativa L.)
Source: Genes (Basel). 2022 Mar 3;13(3):458. doi: 10.3390/genes13030458 (PMC8952259; doi:10.3390/genes13030458)
Supplement: Supplementary file 1 [file genes-13-00458-s001.zip › genes-1597984-supplementary.pdf]

Table S1. Pollen and spikelet fertility of hybrid combinations developed by crossing between IR69625A and G 46A cytoplasmic male sterile lines with new restorer lines during the 2018 season.

Traits crosses	IR69625A		G 46A	
	**Pollen fertility (%)**	**Spikelet fertility (%)**	**Pollen fertility (%)**	**Spikelet fertility (%)**
NRL 63	95.45	91.48	97.27	93.25
NRL 64	97.26	92.11	98.38	94.63
NRL 65	89.23	87.08	96.95	92.34
NRL 66	97.13	92.3	92.88	88.93
NRL 67	96.71	92.82	97.02	93.17
NRL 68	89.62	88.04	94.00	90.11
NRL 69	90.82	88.16	92.23	89.9
NRL 70	93.25	91.88	96.75	91.95
NRL 71	92.83	91.19	97.27	94.67
NRL 72	90.99	88.44	98.38	94.61
NRL 73	97.25	93.86	96.95	92.59
NRL 74	90.9	88.68	92.88	89.83
NRL 26	95.16	90.66	92.89	88.9
NRL 37	89.86	87.66	97.84	92.68
NRL 52	92.14	89.65	97.70	93.12
NRL 53	90.2	87.35	94.20	91.5
NRL 54-2W	91.73	89.65	98.02	92.46
NRL 58	90.83	88.46	96.68	91.3
NRL 59	92.43	87.76	96.73	92.64
NRL 55	94.76	91.38	98.11	93.06
NRL 46	90.76	87.64	90.11	88.87
NRL 62	98.07	95.12	91.55	89.73
G 46A	0	0	0	0
IR69625A	0	0	0	0
86945-L	97.99	92.86	96.25	91.94
BG 33-5	95.45	92.11	97.26	91.48
BG 34-8	97.13	92.82	96.71	92.3

Giza 178	96.42	91.28	95.81	91.32

Table S2: Mean performances for no. of days to 50% heading (day), plant height (cm), number of panicles per plant, panicle length (cm), and number of spikelets per panicle of the studied genotypes during 2019 and 2020 growing season.

Traits crosses	Days to heading		Plant height		No. Of Panicles/plant		Panicle length		No. of spikelets/ panicle	
	2019	2020	2019	2020	2019	2020	2019	2020	2019	2020
NRL 63	102.07	102.70	118.73	119.87	14.5	17.07	24.01	25.27	187.5	192.6
NRL 64	103.10	103.51	119.27	121.63	13.5	15.73	23.05	23.15	181.9	188
NRL 65	102.33	102.47	120.60	123.47	14.5	17.6	23.28	24.04	180.6	186.8
NRL 66	99.07	99.33	101.33	102.07	15.4	18.72	25.4	25.56	187.9	206.9
NRL 67	89.50	90.67	98.00	98.73	16.7	15.9	24.2	24.58	180.6	188.3
NRL 68	89.53	90.00	80.13	81.20	16.4	16.8	21.34	20.3	120.6	114.7
NRL 69	90.47	91.00	82.57	83.73	18.4	18.87	20.88	19.93	110.3	115.5
NRL 70	94.83	95.67	79.47	80.60	16.7	17.27	20.48	19.62	120	115.5
NRL 71	93.20	94.00	80.63	81.40	18.4	19.2	21	20.12	128.6	124
NRL 72	92.50	93.33	79.87	81.00	16.6	17.2	21.13	20.64	127.7	122.9
NRL 73	86.10	87.23	79.67	80.50	18.4	17.37	18.61	20.05	142.3	149.1
NRL 74	85.83	86.47	81.20	81.90	22.9	21.5	18.87	19.73	141.2	148.4
NRL 26	95.60	96.83	118.17	119.50	17.2	18.67	23.66	26.08	224	237.4
NRL 37	99.17	98.17	114.50	117.17	20.8	19.33	27.48	26.28	250.2	229.8
NRL 52	68.30	68.00	96.00	98.33	13.5	14	21.18	20.62	134.2	132.5
NRL 53	75.23	76.23	114.50	116.17	20.5	19	24.06	24.75	188.2	205.3
NRL 54-2W	94.80	95.70	122.33	124.17	15.8	16.67	26.16	26.75	222.8	213.9
NRL 58	99.43	98.50	124.00	122.33	17.7	16.33	25.65	26.38	226.8	215.6
NRL 59	99.83	100.47	122.50	121.17	18.3	16.33	24.5	24.24	187	202.2
NRL 55	100.67	101.50	125.67	123.93	17.3	18.5	26.24	26.42	131.9	145.8
NRL 46	100.90	101.63	115.33	116.50	17.5	18.17	25.66	24.88	170.5	156.2
NRL 62	100.50	99.80	117.67	116.00	20.7	18.97	26.59	26.66	202.2	186.4
G46B	81.00	80.11	91.07	89.45	11.9	12.12	20.93	22.53	175.3	160.2
IR69625B	100.80	100.30	100.40	102.27	15.5	15.77	20.23	22.35	148.4	140.9

IR79156B	103.93	104.73	109.57	110.73	16.3	15.83	24	25.13	204.8	212.5
86945-L	104.23	104.03	111.40	109.80	15.0	14.5	22.45	23.29	153.5	141.3
BG 33-5	104.17	104.03	114.83	113.07	16.0	15.5	23.14	24.73	182.8	159.6
BG 34-8	105.50	106.20	115.17	115.67	14.8	14.17	22.42	22.5	149.9	147.6
Giza 178 (ck)	100.77	100.83	91.00	91.03	12.6	14.13	22.03	23.71	154.2	170.2
L.S.D 5%	0.99	1.00	3.62	4.55	2.4	2.27	1.05	1.16	12.49	10.73
L.S.D 1%	1.32	1.33	4.82	6.06	3.2	3.03	1.4	1.55	16.63	14.28

Table S3: Mean performances for spikelet fertility (%), number of filled grains per panicle, panicle weight (g), 1000 grain weight (g), and grain yield per plant (g) of the studied genotypes during 2019 and 2020 growing season.

Traits crosses	Spikelet fertility (%)		No. of filled grains /panicle		Panicle weight		1000- grain weight		grain yield (g/p)	
	2019	2020	2019	2020	2019	2020	2019	2020	2019	2020
NRL 63	90.17	92.88	169.07	178.90	4.33	5.17	24.77	25.51	52.7	56.52
NRL 64	87.84	90.18	159.80	169.53	4.00	4.67	24.67	26.39	44.13	48.14
NRL 65	92.59	95.44	167.27	178.30	4.31	5.36	25.13	27.04	45.01	47.98
NRL 66	92.06	91.28	172.93	188.80	4.55	5.05	26.33	27.09	51.32	54.02
NRL 67	95.19	96.08	171.80	180.80	4.69	4.27	24.51	25.30	42.36	45.6
NRL 68	91.18	92.44	109.93	106.00	3.72	3.19	28.17	27.43	40.41	42.95
NRL 69	91.83	91.86	101.33	106.10	3.74	3.06	28.75	28.23	49.54	49.76
NRL 70	92.32	92.77	110.73	107.13	3.53	3.05	28.56	27.47	41.58	45.3
NRL 71	92.92	93.69	119.50	116.20	3.68	3.15	28.71	27.67	44.04	47.36
NRL 72	95.29	96.90	121.77	119.17	4.04	3.45	29.06	28.33	38.38	41.36
NRL 73	94.32	93.26	134.47	139.17	3.62	4.11	26.86	27.95	27.6	32.33
NRL 74	95.00	95.55	134.27	141.80	3.49	4.10	26.59	27.73	35.86	39.79
NRL 26	92.29	95.03	206.63	225.60	6.21	6.46	26.97	26.96	38.20	41.00
NRL 37	94.80	92.02	237.17	211.43	6.94	6.48	25.03	24.23	46.05	45.16
NRL 52	96.87	97.13	129.93	128.67	3.83	3.52	29.2	28.93	38.47	41.03
NRL 53	90.36	93.46	169.93	191.83	5.17	5.7	28.47	28.73	45.43	47.37

NRL 54-2W	91.87	92.35	204.60	197.47	6.94	7.08	27.52	28.2	46.94	49.57
NRL 58	88.10	90.35	199.73	194.77	6.30	5.88	27.88	28.23	36.83	40.09
NRL 59	87.00	91.20	162.73	184.43	4.84	5.08	25.83	25.6	56.82	60.23
NRL 55	88.03	92.46	116.07	134.77	4.28	4.48	32.27	32.02	55.43	58.55
NRL 46	90.74	92.10	154.73	143.90	5.28	5.02	29.13	30.16	48.84	51.83
NRL 62	91.49	94.49	184.93	176.10	5.92	5.61	27.23	27.9	53.80	56.97
G46B	88.37	90.72	154.97	145.33	4.10	3.42	24.4	24.43	30.50	34.10
IR69625B	90.61	91.40	134.43	128.80	4.02	3.80	23.99	24.57	37.27	39.52
IR79156B	90.16	90.72	184.67	192.70	3.81	3.69	21.13	21.27	35.74	38.47
86945-L	89.68	91.37	137.60	129.03	3.37	4.07	25.26	26.38	36.09	39.40
BG 33-5	91.70	94.06	167.60	150.13	3.88	5.21	26.14	26.64	32.67	35.35
BG 34-8	88.57	90.24	132.77	133.20	3.82	3.79	26.74	27.21	31.43	34.11
Giza 178 (ck)	91.61	91.68	141.23	156.00	2.82	3.42	21.3	20.46	36.83	39.17
L.S.D 5%	2.40	1.58	12.14	9.95	0.48	0.54	0.69	0.60	4.58	4.10
L.S.D 1%	3.20	2.11	16.16	13.25	0.64	0.72	0.92	0.79	6.1	5.45

Table S4: Mean performances for floral traits of the studied genotypes during 2019 and 2020 growing season.

Traits crosses	Anther length (mm)		Anther breadth (mm)		Filament length (mm)		Duration of floret opening (min)	
	2019	2020	2019	2020	2019	2020	2019	2020
NRL 63	2.42	2.37	0.47	0.46	7.03	7.17	128.64	127.31
NRL 64	2.01	1.97	0.45	0.43	6.96	6.92	120.16	118.77
NRL 65	1.97	1.91	0.46	0.44	6.83	6.77	114.33	113.02
NRL 66	2.12	2.07	0.53	0.51	7.47	7.41	133.12	131.68
NRL 67	2.22	2.16	0.46	0.44	6.47	6.40	131.23	129.36
NRL 68	2.30	2.25	0.42	0.41	6.47	6.41	131.18	129.32
NRL 69	2.23	2.17	0.44	0.45	6.63	6.53	118.24	120.24
NRL 70	2.14	2.09	0.44	0.45	6.27	6.19	116.20	114.54
NRL 71	2.22	2.17	0.46	0.45	6.57	6.47	130.48	129.48
NRL 72	2.39	2.34	0.46	0.44	6.83	6.70	129.78	128.83
NRL 73	2.23	2.19	0.48	0.46	6.70	6.58	129.39	130.70

NRL 74	2.34	2.28	0.46	0.47	6.70	6.59	130.14	131.49
NRL 26	2.80	2.84	0.55	0.57	7.30	7.44	154.82	153.53
NRL 37	2.75	2.78	0.54	0.54	7.20	7.29	153.07	152.03
NRL 52	2.03	2.00	0.41	0.41	6.27	6.13	139.14	140.13
NRL 53	2.13	2.07	0.44	0.44	6.13	5.97	137.61	138.88
NRL 54-2W	2.92	2.95	0.59	0.60	7.60	7.70	157.72	156.60
NRL 58	2.81	2.77	0.56	0.58	7.33	7.47	151.48	152.44
NRL 59	2.74	2.71	0.55	0.54	7.17	7.03	152.62	153.82
NRL 55	2.64	2.66	0.45	0.45	7.03	7.17	129.05	127.73
NRL 46	2.72	2.76	0.43	0.42	7.13	7.03	128.88	128.04
NRL 62	2.85	2.87	0.57	0.56	7.47	7.34	148.86	148.10
G46B	1.80	1.83	0.34	0.35	6.30	6.24	154.32	152.82
IR69625B	1.83	1.85	0.37	0.38	6.10	6.02	150.63	149.42
IR79156B	1.91	1.94	0.31	0.33	6.40	6.37	144.56	143.33
86945-L	1.75	1.76	0.39	0.39	6.20	6.13	92.90	91.67
BG 33-5	1.82	1.84	0.37	0.38	6.17	6.03	94.02	92.99
BG 34-8	1.90	1.93	0.36	0.37	6.27	6.13	95.20	94.06
Giza 178 (ck)	1.78	1.79	0.41	0.42	4.30	4.20	76.39	77.53
L.S.D 5%	0.29	0.29	0.03	0.03	0.56	0.57	5.04	4.42
L.S.D 1%	0.38	0.38	0.04	0.04	0.75	0.75	6.71	5.88

Table S5: Mean performances for rice hulling (%), milling (%), and head rice percentage traits of the studied genotypes during the 2019 and 2020 growing season.

Traits crosses	Hulling (%)		Milling (%)		Head rice %	
	2019	2020	2019	2020	2019	2020
NRL 63	77.8	78.56	66.93	70.32	40.06	41.47
NRL 64	77.87	78.96	69	71.59	38.8	39.3
NRL 65	79.07	79.38	68.4	73.01	43.07	46.23
NRL 66	79.4	79.27	70.87	71.49	63.33	65.63
NRL 67	79.04	79.79	71.49	70.69	68.4	67.78
NRL 68	80.13	80.67	72.28	73.33	52.86	53.33
NRL 69	78.92	79.27	69.5	70	40.65	41
NRL 70	80.41	81	71.51	72.6	62.05	62.6
NRL 71	79.86	80.53	70.09	71.2	48.61	49.2
NRL 72	79.99	80.6	69.83	70.73	58.97	59.53
NRL 73	79.24	79.98	72.71	71.57	51.67	52.66
NRL 74	78.98	79.57	72.22	71.64	61.33	61.64
NRL 26	81.33	81.88	71.09	71.44	51.55	52.51
NRL 37	81.6	80.32	69.27	70.67	61.47	64.67
NRL 52	82.4	83.33	72.03	70.86	67.67	68.67
NRL 53	81.13	81.52	72.4	71.34	68.4	67.5
NRL 54-2W	79.83	79.4	70.46	69.97	64.02	63.67
NRL 58	81.33	81.67	70.33	69.46	56.68	55.86
NRL 59	79.47	79.51	72.27	72.27	70.88	70.37
NRL 55	81.6	81.38	75	74.18	67.33	67.1
NRL 46	80.8	81.73	72.8	73.13	70.07	71
NRL 62	81.8	80.34	71.93	72.28	64.39	65.77
G46B	80.16	80.53	70.89	69.87	49.81	48.27
IR69625B	80.25	79.67	72.67	70.82	60.77	58.34
IR79156B	80.58	80.2	70.28	69.48	57.3	56.08
86945-L	79.17	80.38	69.8	70.87	59.07	58.15
BG 33-5	79.64	80.63	71.37	70.93	56.34	55.28
BG 34-8	79.67	80.43	71.7	71.72	63.2	62.08

Giza 178 (ck)	81.04	81.37	71.01	71.88	66.68	66.68
L.S.D 5%	0.97	1.12	1.01	1.46	2.32	4.28
L.S.D 1%	1.29	1.49	1.34	1.94	3.09	5.69

Table S6: Mean performances for resistance to blast disease and rice stem borer insect during the 2019 and 2020 growing season.

	Blast disease		stem borer insect	
Lines	2019	2020	2019	2020
NRL 63	R	R	R	R
NRL 64	R	R	R	R
NRL 65	R	R	R	R
NRL 66	R	R	R	R
NRL 67	R	R	R	R
NRL 68	R	R	R	R
NRL 69	R	R	R	R
NRL 70	R	R	R	R
NRL 71	R	R	R	R
NRL 72	R	R	R	R
NRL 73	R	R	R	R
NRL 74	R	R	R	R
NRL 26	R	R	R	R
NRL 37	R	R	R	R
NRL 52	R	R	R	R
NRL 53	R	R	R	R
NRL – 54-2W	R	R	R	R
NRL 58	R	R	R	R
NRL 59	R	R	R	R
NRL 55	R	R	R	R
NRL 46	R	R	R	R
NRL 62	R	R	R	R
G46A, B	R	R	R	R
IR69625A, B	R	R	R	R
IR79156A, B	R	R	R	R

86945-L	R	R	R	R
BG 33-5	R	R	R	R
BG 34-8	R	R	R	R
Giza 178 (ck)	R	R	MR	MR

R : resistance
MR : moderate resistance

Table S7: Estimates of the percentage of advantage over commercial variety and over better parent for days to heading, plant height, and no. of panicles/plant of studied genotypes during the 2019 and 2020 growing season.

Traits crosses	Days to heading				Plant height				No. Of Panicles/plant			
	Advantage over (B.P)		Advantage over Ck		Advantage over (B.P)		Advantage over Ck		Advantage over (B.P)		Advantage over Ck	
	2019	2020	2019	2020	2019	2020	2019	2020	2019	2020	2019	2020
NRL 63	-1.8**	-1.3*	1.3*	1.9**	8.4**	9.2**	30.5**	31.7**	-11.1 ns	7.8 ns	14.5 ns	20.8*
NRL 64	-0.8 ns	-0.5 ns	2.3**	2.7**	8.9**	10.8**	31.1**	33.6**	-17.0*	-0.6 ns	6.9 ns	11.4 ns
NRL 65	-1.5**	-1.5**	1.6**	1.6**	10.1**	12.4**	32.5**	35.6**	-10.7 ns	11.2 ns	15.0 ns	24.6*
NRL 66	22.3**	24.0**	-1.7**	-1.5*	11.4**	14.1**	11.4**	12.1**	21.6 ns	32.5**	21.6 ns	32.5**
NRL 67	10.5**	13.2**	-11.2**	-10.1**	7.7**	10.4**	7.7**	8.5**	31.9**	12.6 ns	31.9**	12.6 ns
NRL 68	10.5**	12.3**	-11.1**	-10.7**	-12.0**	-9.2**	-11.9**	-10.8**	2.3 ns	8.4 ns	29.6**	18.9*
NRL 69	11.7**	13.6**	-10.2**	-9.8**	-9.3**	-6.4*	-9.3**	-8.0**	14.8 ns	21.7*	45.4**	33.6**
NRL 70	17.1**	19.4**	-5.9**	-5.1**	-12.7**	-9.9**	-12.7**	-11.5**	4.6 ns	11.4 ns	32.5**	22.2*
NRL 71	15.1**	17.3**	-7.5**	-6.8**	-11.5**	-9.0**	-11.4**	-10.6**	15.0 ns	23.9**	45.6**	35.9**
NRL 72	14.2**	16.5**	-8.2**	-7.4**	-12.3**	-9.4**	-12.2**	-11.0**	3.5 ns	11.0 ns	31.1**	21.8*
NRL 73	6.3**	8.9**	-14.6**	-13.5**	-12.5**	-10.0**	-12.5**	-11.6**	15.0 ns	12.0 ns	45.6**	22.9*
NRL 74	6.0**	7.9**	-14.8**	-14.2**	-10.8**	-8.4**	-10.8**	-10.0**	43.3**	38.7**	81.5**	52.2**
NRL 26	18.0**	20.9**	-5.1**	-4.0**	29.8**	33.6**	29.9**	31.3**	7.3 ns	20.4*	35.9**	32.1**
NRL 37	22.4**	22.5**	-1.6**	-2.6**	25.7**	31.0**	25.8**	28.7**	30.2**	24.7**	64.9**	36.9**
NRL 52	-15.7**	-15.1**	-32.2**	-32.6**	5.4*	9.9**	5.5*	8.0**	-10.0 ns	-3.4 ns	6.9 ns	-0.9 ns
NRL 53	-7.1**	-4.8**	-25.3**	-24.4**	25.7**	29.9**	25.8**	27.6**	36.7**	31.0**	62.3**	34.5**

NRL 54-2W	17.0**	19.5**	-5.9**	-5.1**	34.3**	38.8**	34.4**	36.4**	6.7 ns	17.6 ns	25.3*	18.0 ns
NRL 58	22.8**	23.0**	-1.3*	-2.3**	36.2**	36.8**	36.3**	34.4**	19.1*	15.3 ns	39.8**	15.6 ns
NRL 59	23.3**	25.4**	-0.9 ns	-0.4 ns	34.5**	35.5**	34.6**	33.1**	23.6*	15.3 ns	45.1**	15.6 ns
NRL 55	-0.1 ns	1.2*	-0.1 ns	0.7 ns	25.2**	21.2**	38.1**	36.1**	12.1 ns	17.3*	37.2**	31.0**
NRL 46	0.1 ns	1.3*	0.1 ns	0.8 ns	14.9**	13.9**	26.7**	28.0**	13.1 ns	15.2 ns	38.5**	28.6**
NRL 62	-0.3 ns	-0.5 ns	-0.3 ns	-1.0 ns	17.2**	13.4**	29.3**	27.4**	33.6**	20.2*	63.6**	34.3**
L.S.D 5%	1.148	1.153	1.148	1.153	4.18	5.26	4.18	5.26	2.74	2.62	2.74	2.62
L.S.D 1%	1.528	1.535	1.528	1.535	5.57	7.00	5.57	7.00	3.65	3.49	3.65	3.49

**: Highly significant at 1% *: Significant at 5% ns: Non-significant L.S.D. : The least significant difference

Table S8: Estimates of the percentage of advantage over commercial variety and over better parent for panicle length, no. of spikelets/panicle, and spikelet fertility percentage of studied genotypes during the 2019 and 2020 growing season.

Traits crosses	Panicle length				No. of spikelets/ panicle				Spikelet fertility (%)			
	Advantage over (B.P)		Advantage over Ck		Advantage over (B.P)		Advantage over Ck		Advantage over (B.P)		Advantage over Ck	
	2019	2020	2019	2020	2019	2020	2019	2020	2019	2020	2019	2020
NRL 63	0.03 ns	0.6 ns	9.0**	6.6*	-8.4*	-9.3**	21.6**	13.1**	0.01ns	1.7ns	-1.6ns	1.3ns
NRL 64	-4.0 ns	-7.9**	4.6 ns	-2.3 ns	-11.2**	-11.5**	18.0**	10.4**	-2.6ns	-1.3ns	-4.1**	-1.6ns
NRL 65	-3.0 ns	-4.3 ns	5.7*	1.4 ns	-11.8**	-12.1**	17.2**	9.8**	2.7ns	4.5**	1.1ns	4.1**
NRL 66	15.3**	7.8**	15.3**	7.8**	7.2 ns	21.5**	21.9**	21.5**	0.5ns	-0.4ns	0.5	-0.4ns
NRL 67	9.9**	3.7 ns	9.9**	3.7 ns	3.0 ns	10.6**	17.2**	10.6**	3.9*	4.8**	3.9*	4.8**
NRL 68	-7.8**	-17.9**	-3.1 ns	-14.4**	-34.0**	-28.4**	-21.8**	-32.6**	-0.6ns	-1.7ns	-0.5ns	0.8ns
NRL 69	-9.8**	-19.4**	-5.2 ns	-16.0**	-39.6**	-27.9**	-28.4**	-32.1**	0.1ns	-2.3*	0.2ns	0.2ns
NRL 70	-11.5**	-20.7**	-7.1*	-17.3**	-34.3**	-27.9**	-22.1**	-32.1**	0.7ns	-1.4ns	0.8ns	1.2ns
NRL 71	-9.3**	-18.7**	-4.7 ns	-15.1**	-29.6**	-22.6**	-16.6**	-27.2**	1.3ns	-0.4ns	1.4ns	2.2*
NRL 72	-8.7**	-16.5**	-4.1 ns	-12.9**	-30.1**	-23.3**	-17.2**	-27.8**	3.9*	3.0**	4.0*	5.7**
NRL 73	-19.6**	-18.9**	-15.5**	-15.4**	-22.1**	-6.9 ns	-7.7 ns	-12.4**	2.9ns	-0.9ns	3.0ns	1.7ns

NRL 74	-18.5**	-20.2**	-14.4**	-16.8**	-22.8**	-7.3 ns	-8.4 ns	-12.8**	3.6*	1.6ns	3.7*	4.2**
NRL 26	2.3 ns	5.4*	7.4**	10.0**	22.6**	48.2**	45.3**	39.5**	0.6ns	1.0ns	0.7ns	3.7**
NRL 37	18.8**	6.3*	24.7**	10.8**	36.9**	43.4**	62.3**	35.0**	3.4*	-2.2*	3.5*	0.4ns
NRL 52	-5.6*	-11.5**	-3.9 ns	-13.0**	-23.5**	-17.3**	-12.9**	-22.2**	8.0**	6.3**	5.7**	5.9**
NRL 53	7.2*	6.3*	9.2**	4.4 ns	7.3 ns	28.1**	22.1**	20.6**	0.8ns	2.3*	-1.4ns	1.9ns
NRL 54-2W	16.7**	18.7**	18.7**	12.8**	27.1**	33.5**	44.5**	25.6**	3.7*	1.8ns	0.3ns	0.7ns
NRL 58	14.4**	17.1**	16.4**	11.3**	29.4**	34.6**	47.1**	26.6**	-0.5ns	-0.4ns	-3.8*	-1.4ns
NRL 59	9.3**	7.6*	11.2**	2.2 ns	6.7 ns	26.2**	21.3**	18.8**	-1.8ns	0.5ns	-5.0**	-0.5ns
NRL 55	17.0**	17.4**	19.1**	11.4**	-12.0*	-1.2 ns	-14.5**	-14.4**	-2.9ns	1.2ns	-3.9*	0.9ns
NRL 46	14.4**	10.6**	16.5**	4.9 ns	13.7**	5.8 ns	10.6*	-8.2*	0.1ns	0.8ns	-1.0ns	0.5ns
NRL 62	18.6**	18.5**	20.7**	12.4**	34.9**	26.3**	31.2**	9.5*	1.0ns	3.4**	-0.1ns	3.1**
L.S.D 5%	1.22	1.34	1.22	1.34	14.43	12.39	14.43	12.39	2.77	1.83	2.77	1.83
L.S.D 1%	1.62	1.79	1.62	1.79	19.20	16.49	19.20	16.49	3.69	2.43	3.69	2.43

**: Highly significant at 1% *: Significant at 5% ns: Non-significant L.S.D. : The least significant difference

Table S9: Estimates of the percentage of advantage over commercial variety and over better parent for No. of filled grains/panicle, panicle weight, and 1000-grain weight of studied genotypes during the 2019 and 2020 growing season.

Traits crosses	No. of filled grains/panicle				Panicle weight				1000- grain weight			
	Advantage over (B.P)		Advantage over Ck		Advantage over (B.P)		Advantage over Ck		Advantage over (B.P)		Advantage over Ck	
	2019	2020	2019	2020	2019	2020	2019	2020	2019	2020	2019	2020
NRL 63	-8.4*	-7.2*	19.7**	14.7**	13.7 ns	27.0**	53.5**	51.4**	-1.9 ns	-3.3*	16.3**	24.7**
NRL 64	-13.5**	-12.0**	7.6 ns	8.7*	5.0 ns	14.6 ns	41.7**	36.7**	-2.3 ns	0.01 ns	15.8**	28.9**
NRL 65	-9.4*	-7.5*	18.4**	14.3**	13.0 ns	31.6**	52.5**	56.9**	-0.5 ns	2.5 ns	18.0**	32.1**
NRL 66	11.6*	21.0**	22.4**	21.0**	11.0 ns	47.8**	61.0**	47.9**	7.9**	10.9**	23.6**	32.4**
NRL 67	10.9*	15.9**	21.6**	15.9**	14.6*	25.0**	66.2**	25.1**	0.5 ns	3.6*	15.1**	23.7**
NRL 68	-34.4**	-29.4**	-22.2**	-32.1**	-9.3 ns	-38.7**	31.6**	-6.5 ns	7.8**	3.0*	32.3**	34.1**
NRL 69	-39.5**	-29.3**	-28.3**	-32.0**	-8.7 ns	-41.3**	32.5**	-10.4 ns	10.0**	6.0**	35.0**	38.0**

NRL 70	-33.9**	-28.6**	-21.6**	-31.4**	-13.8*	-41.4**	25.0*	-10.6 ns	9.2**	3.1*	34.1**	34.2**
NRL 71	-28.7**	-22.6**	-15.4**	-25.5**	-10.3 ns	-39.5**	30.2**	-7.7 ns	9.8**	3.9**	34.8**	35.2**
NRL 72	-27.3**	-20.6 **	-13.8**	-23.6**	-1.4 ns	-33.9**	43.1**	0.9 ns	11.2**	6.4**	36.4**	38.5**
NRL 73	-19.8**	-7.3ns	-4.8 ns	-10.8**	-11.7 ns	-21.1**	28.1**	20.4*	2.7 ns	4.9**	26.1**	36.6**
NRL 74	-19.9**	-5.6ns	-4.9 ns	-9.1*	-14.7*	-21.4**	23.7*	20.0*	1.7 ns	4.1**	24.9**	35.5**
NRL 26	23.3**	50.3**	46.3**	44.6**	51.6**	23.9**	120.0**	89.1**	3.2*	1.2 ns	26.6**	31.7**
NRL 37	41.5**	40.8**	67.9**	35.5**	69.4**	24.3**	145.8**	89.7**	-4.3**	-9.0**	17.5**	18.4**
NRL 52	-16.2**	-11.5**	-8.0ns	-17.5**	-6.5 ns	-13.6 ns	35.7**	3.0 ns	15.6**	9.7**	37.1**	41.4**
NRL 53	9.7*	32.0**	20.3**	23.0**	26.2**	39.9**	83.1**	66.8**	12.7**	8.9**	33.6**	40.4**
NRL 54-2W	32.0**	35.9**	44.9**	26.6**	69.4**	86.7**	145.8**	107.3**	2.9 ns	3.6**	29.2**	37.8**
NRL 58	28.9**	34.0**	41.4**	24.9**	53.8**	55.0**	123.1**	72.1**	4.3**	3.7**	30.9**	38.0**
NRL 59	5.0 ns	26.9**	15.2**	18.2**	18.1**	33.9**	71.4**	48.7**	-3.4*	-5.9**	21.3**	25.1**
NRL 55	-13.7*	1.2ns	-17.8**	-13.6**	6.5 ns	17.9*	51.6**	31.1**	20.7**	17.7**	51.5**	56.5**
NRL 46	15.1**	8.0ns	9.6ns	-7.8*	31.3**	32.1**	87.0**	46.9**	9.0**	10.8**	36.8**	47.4**
NRL 62	37.6**	32.2**	30.9**	12.9**	47.3**	47.6**	109.7**	64.2**	1.9 ns	2.5 ns	27.9**	36.3**
L.S.D 5%	14.02	11.49	14.02	11.49	0.55	0.62	0.55	0.62	0.80	0.69	0.80	0.69
L.S.D 1%	18.66	15.30	18.66	15.30	0.74	0.83	0.74	0.83	1.06	0.92	1.06	0.92

**: Highly significant at 1% *: Significant at 5% ns: Non-significant L.S.D. : The least significant difference

Table S10: Estimates of the percentage of advantage over commercial variety and over better parent for grain yield, anther length, and anther breadth of studied genotypes during the 2019 and 2020 growing season.

Traits crosses	grain yield (gm/P)				Anther length (mm)				Anther breadth (mm)			
	Advantage over (B.P)		Advantage over Ck		Advantage over (B.P)		Advantage over Ck		Advantage over (B.P)		Advantage over Ck	
	2019	2020	2019	2020	2019	2020	2019	2020	2019	2020	2019	2020

RL 63	46.0**	43.4**	43.1**	44.3**	26.66**	22.55*	36.1**	32.6**	22.41**	16.10**	16.4**	8.7*
NRL 64	22.3**	22.2**	19.8**	22.9**	5.2ns	1.7ns	13.1ns	10.1ns	17.2**	10.2*	11.5**	3.2ns
NRL 65	24.7**	21.8**	22.2**	22.5**	2.8ns	-1.5ns	10.5ns	6.5ns	19.8**	12.7**	13.9**	5.6ns
NRL 66	39.3**	37.9**	39.3**	37.9**	17.6ns	13.1ns	18.9*	15.5ns	29.5**	22.2**	29.5**	22.2**
NRL 67	15.0*	16.4**	15.0*	16.4**	23.1*	18.1ns	24.5*	20.5*	12.3**	4.0ns	12.3**	4.0ns
NRL 68	23.7**	21.5**	9.7 ns	9.7 ns	26.8**	22.7*	29.4**	25.9**	12.5**	6.1ns	3.3ns	-3.2ns
NRL 69	51.7**	40.8**	34.5**	27.0**	22.8*	18.0ns	25.3**	21.0*	17.0**	17.4**	7.4ns	7.1ns
NRL 70	27.3**	28.2**	12.9 ns	15.7*	17.8ns	14.0ns	20.2*	16.9ns	18.8**	18.3**	9.0*	7.9*
NRL 71	34.8**	34.0**	19.6**	20.9**	22.4*	18.3*	24.9**	21.4*	24.1**	18.3**	13.9**	7.9*
NRL 72	17.5*	17.0*	4.2 ns	5.6 ns	31.6**	27.4**	34.3**	30.7**	22.3**	14.8**	12.3**	4.8ns
NRL 73	-15.5 ns	-8.5 ns	-25.1**	-17.5**	22.9*	19.1*	25.5**	22.2*	29.5**	20.9**	18.9**	10.3**
NRL 74	9.8 ns	12.6 ns	-2.6 ns	1.6 ns	29.0**	24.3**	31.6**	27.6**	22.3**	21.7**	12.3**	11.1**
NRL 26	16.9*	16.0*	3.7 ns	4.7 ns	54.1**	54.8**	57.3**	58.8**	47.3**	47.8**	35.2**	34.9**
NRL 37	41.0**	27.8**	25.0**	15.3*	51.6**	51.4**	54.7**	55.3**	45.5**	41.7**	33.6**	29.4**
NRL 52	6.6 ns	4.1 ns	4.5 ns	4.8 ns	12.8ns	9.7ns	14.0ns	11.9ns	6.9ns	5.1ns	1.6ns	-1.6ns
NRL 53	25.9**	20.2**	23.3**	20.9**	18.3ns	13.3ns	19.7*	15.6ns	12.9**	11.0**	7.4ns	4.0ns
NRL 54-2W	49.3**	45.3**	27.4**	26.6**	53.9**	52.9**	64.2**	64.6**	63.3**	60.7**	45.9**	42.9**
NRL 58	17.2*	17.5*	0.0 ns	2.4 ns	47.9**	43.6**	57.9**	54.6**	55.0**	55.4**	38.5**	38.1**
NRL 59	80.8**	76.6**	54.3**	53.8**	44.4**	40.7**	54.1**	51.4**	51.4**	44.6**	35.2**	28.6**
NRL 55	48.7**	48.1**	50.5**	49.5**	38.8**	38.1**	48.1**	48.6**	21.6**	17.4**	10.7*	7.1ns
NRL 46	31.0**	31.2**	32.6**	32.3**	43.3**	43.1**	53.0**	54.0**	16.2**	9.6*	5.7ns	0.0ns
NRL 62	44.3**	44.1**	46.1**	45.4**	49.8**	48.8**	59.9**	60.1**	54.1**	45.2**	40.2**	32.5**
L.S.D 5%	5.29	4.73	5.29	4.73	0.33	0.33	0.33	0.33	0.03	0.03	0.03	0.03
L.S.D 1%	7.04	6.30	7.04	6.30	0.44	0.44	0.44	0.44	0.05	0.04	0.05	0.04

**: Highly significant at 1% *: Significant at 5% ns: Non-significant L.S.D. : The least significant difference

Table S11: Estimates of the percentage of advantage over commercial variety and over better parent for filament length and duration of floret opening of studied genotypes during the 2019 and 2020 growing season.

Traits crosses	Filament length (mm)				Duration of floret opening (min)			
	Advantage over (B.P)		Advantage over Ck		Advantage over (B.P)		Advantage over Ck	
	2019	2020	2019	2020	2019	2020	2019	2020
RL 63	9.90ns	12.57*	63.6**	70.6**	-11.01**	-11.17**	68.4**	64.2**
NRL 64	8.7ns	8.7ns	61.9**	64.8**	-16.9**	-17.1**	57.3**	53.2**
NRL 65	6.8ns	6.3ns	58.9**	61.1**	-20.9**	-21.1**	49.7**	45.8**
NRL 66	18.5**	18.7**	73.6**	76.5**	-13.7**	-13.8**	74.3**	69.8**
NRL 67	2.6ns	2.6ns	50.4**	52.5**	-15.0**	-15.4**	71.8**	66.9**
NRL 68	2.6ns	2.7ns	50.4**	52.7**	-15.0**	-15.4**	71.7**	66.8**
NRL 69	5.3ns	4.6ns	54.3**	55.5**	-23.4**	-21.3**	54.8**	55.1**
NRL 70	-0.5ns	-0.9ns	45.7**	47.4**	-24.7**	-25.1**	52.1**	47.7**
NRL 71	4.2ns	3.6ns	52.7**	54.0**	-15.5**	-15.3**	70.8**	67.0**
NRL 72	8.5ns	7.3ns	58.9**	59.5**	-15.9**	-15.7**	69.9**	66.2**
NRL 73	6.3ns	5.4ns	55.8**	56.7**	-16.2**	-14.5**	69.4**	68.6**
NRL 74	6.3ns	5.5ns	55.8**	56.8**	-15.7**	-14.0**	70.4**	69.6**
NRL 26	15.9**	19.2**	69.8**	77.1**	0.3ns	0.5ns	102.7**	98.0**
NRL 37	14.3**	16.7**	67.4**	73.5**	-0.8ns	-0.5ns	100.4**	96.1**
NRL 52	-0.5ns	-1.8ns	45.7**	46.0**	-9.8**	-8.3**	82.1**	80.7**
NRL 53	-2.6ns	-4.4ns	42.6**	42.1**	-10.8**	-9.1**	80.1**	79.1**
NRL 54-2W	20.6**	23.4**	76.7**	83.4**	2.2ns	2.5ns	106.5**	102.0**
NRL 58	16.4**	19.6**	70.5**	77.8**	-1.8ns	-0.2ns	98.3**	96.6**
NRL 59	13.8*	12.7*	66.7**	67.5**	-1.1ns	0.7ns	99.8**	98.4**
NRL 55	12.2*	16.8**	63.6**	70.6**	-14.3**	-14.5**	68.9**	64.8**
NRL 46	13.8*	14.7**	65.9**	67.5**	-14.4**	-14.3**	68.7**	65.2**
NRL 62	19.1**	19.7**	73.6**	74.8**	-1.2ns	-0.9ns	94.9**	91.0**
L.S.D 5%	0.65	0.65	0.65	0.65	5.82	5.10	5.82	5.10
L.S.D 1%	0.87	0.87	0.87	0.87	7.75	6.79	7.75	6.79

**: Highly significant at 1% *: Significant at 5% ns: Non-significant L.S.D. : The least significant difference

Table S12: Estimates of the percentage of advantage over commercial variety and over better parent for some grain quality traits of studied genotypes during the 2019 and 2020 growing season.

Traits crosses	Hulling (%)				Milling (%)				Head rice %			
	Advantage over (B.P)		Advantage over Ck		Advantage over (B.P)		Advantage over Ck		Advantage over (B.P)		Advantage over Ck	
	2019	2020	2019	2020	2019	2020	2019	2020	2019	2020	2019	2020
RL 63	-3.5**	-2.3**	-4.0**	-3.5**	-4.8**	-0.8 ns	-5.7**	-2.2 ns	-32.2**	-28.7**	-39.9**	-37.8**
NRL 64	-3.4**	-1.8*	-3.9**	-3.0**	-1.8*	1.0 ns	-2.8**	-0.4 ns	-34.3**	-32.4**	-41.8**	-41.1**
NRL 65	-1.9**	-1.2 ns	-2.4**	-2.4**	-2.7**	3.0*	-3.7**	1.6 ns	-27.1**	-20.5**	-35.4**	-30.7**
NRL 66	-2.0**	-2.6**	-2.0**	-2.6**	-0.2 ns	-0.5 ns	-0.2 ns	-0.5 ns	-5.0*	-1.6 ns	-5.0*	-1.6 ns
NRL 67	-2.5**	-1.9*	-2.5**	-1.9*	0.7 ns	-1.7 ns	0.7 ns	-1.7 ns	2.6 ns	1.6 ns	2.6 ns	1.6 ns
NRL 68	0.04 ns	0.05 ns	-1.1 ns	-0.9 ns	1.3 ns	3.4**	1.8*	2.0 ns	-6.2*	-3.5 ns	-20.7**	-20.0**
NRL 69	-1.5*	-1.7*	-2.6**	-2.6**	-2.6**	-1.3 ns	-2.1*	-2.6*	-27.9**	-25.8**	-39.0**	-38.5**
NRL 70	0.3 ns	0.5 ns	-0.8 ns	-0.5 ns	0.2 ns	2.4 ns	0.7 ns	1.0 ns	10.1**	13.2**	-6.9**	-6.1 ns
NRL 71	-0.4 ns	-0.1 ns	-1.5*	-1.0 ns	-1.8*	0.4 ns	-1.3 ns	-0.9 ns	-13.7**	-11.0*	-27.1**	-26.2**
NRL 72	-0.2 ns	0.04 ns	-1.3 ns	-0.9 ns	-2.2*	-0.3 ns	-1.7*	-1.6 ns	4.7 ns	7.7 ns	-11.6**	-10.7**
NRL 73	-1.1 ns	-0.8 ns	-2.2**	-1.7*	1.9*	0.9 ns	2.4**	-0.4 ns	-8.3**	-4.7 ns	-22.5**	-21.0**
NRL 74	-1.5*	-1.3 ns	-2.5**	-2.2**	1.2 ns	1.0 ns	1.7*	-0.3 ns	8.9**	11.5*	-8.0**	-7.6*
NRL 26	1.5*	1.6 ns	0.4 ns	0.6 ns	-0.4 ns	0.7 ns	0.1 ns	-0.6 ns	-8.5**	-5.0 ns	-22.7**	-21.3**
NRL 37	1.8*	-0.4 ns	0.7 ns	-1.3 ns	-2.9**	-0.4 ns	-2.4**	-1.7 ns	9.1**	17.0**	-7.8**	-3.0 ns
NRL 52	2.8**	3.5**	1.7*	2.4**	1.6 ns	0.0 ns	1.4 ns	-1.4 ns	14.6**	18.1**	1.5 ns	3.0 ns
NRL 53	1.2 ns	1.2 ns	0.1 ns	0.2 ns	2.1*	0.7 ns	2.0*	-0.7 ns	15.8**	16.1**	2.6 ns	1.2 ns
NRL 54-2W	-0.4 ns	-1.4 ns	-1.5*	-2.4**	-1.7*	-2.4*	-0.8 ns	-2.7*	1.3 ns	2.6 ns	-4.0 ns	-4.5 ns
NRL 58	1.5*	1.4 ns	0.4 ns	0.4 ns	-1.9*	-3.1**	-1.0 ns	-3.4**	-10.3**	-10.0*	-15.0**	-16.2**
NRL 59	-0.9 ns	-1.3 ns	-1.9**	-2.3**	0.8 ns	0.8 ns	1.8*	0.5 ns	12.1**	13.3**	6.3**	5.5 ns
NRL 55	1.7*	1.2 ns	0.7 ns	0.01 ns	3.2**	3.4**	5.6**	3.2**	6.5**	8.1*	1.0 ns	0.6 ns
NRL 46	0.7 ns	1.6*	-0.3 ns	0.4 ns	0.2 ns	2.0 ns	2.5**	1.7 ns	10.9**	14.4**	5.1*	6.5 ns
NRL 62	1.9**	-0.1 ns	0.9 ns	-1.3 ns	-1.0 ns	0.8 ns	1.3 ns	0.6 ns	1.9 ns	5.9 ns	-3.4 ns	-1.4 ns
L.S.D 5%	1.12	1.29	1.12	1.29	1.16	1.68	1.16	1.68	2.68	4.94	2.68	4.94
L.S.D 1%	1.49	1.72	1.49	1.72	1.55	2.24	1.55	2.24	3.56	6.57	3.56	6.57

**: Highly significant at 1% *: Significant at 5% ns: Non-significant L.S.D. : The least significant difference
